# Review on Optical Imaging Techniques for Multispectral Analysis of Nanomaterials

**DOI:** 10.7150/ntno.63222

**Published:** 2022-01-01

**Authors:** Haeni Lee, Jaeheung Kim, Hyung-Hoi Kim, Chang-Seok Kim, Jeesu Kim

**Affiliations:** 1 Department of Cogno-Mechatronics Engineering, College of Nanoscience & Nanotechnology, Pusan National University, Busan 46241, Republic of Korea.; 2 Department of Laboratory Medicine and Biomedical Research Institute, Pusan National University Hospital and Pusan National University School of Medicine, Busan 49241, Republic of Korea.

**Keywords:** Multispectral imaging, Nanoparticles, Spectral unmixing, Contrast-enhanced imaging, Biomedical imaging

## Abstract

Biomedical imaging is an essential tool for investigating biological responses *in vivo*. Among the several imaging techniques, optical imaging systems with multispectral analysis of nanoparticles have been widely investigated due to their ability to distinguish the substances in biological tissues *in vivo*. This review article focus on multispectral optical imaging techniques that can provide molecular functional information. We summarize the basic principle of the spectral unmixing technique that enables the delineation of optical chromophores. Then, we explore the principle, typical system configuration, and biomedical applications of the representative optical imaging techniques, which are fluorescence imaging, two-photon microscopy, and photoacoustic imaging. The results in the recent studies show the great potential of the multispectral analysis techniques for monitoring responses of biological systems *in vivo*.

## Introduction

Biomedical imaging is an efficient way to investigate the physiological and morphological responses of biological cells, tissues, or organs. Especially, preclinical small animal studies have widely been conducted by visualizing the molecular responses, structural deformation, or material distribution *in vivo* 1. Several biomedical imaging techniques such as X-ray computed tomography (CT) 2-4, magnetic resonance imaging (MRI) 5, 6, positron emission tomography (PET) 5, 6, single-photon emission computed tomography (SPECT) 7, 8, ultrasound imaging (USI) 9-11, and optical imaging 12 have been used for preclinical small animal studies. Among the biomedical imaging modalities, optical imaging techniques have been widely used due to their strong optical contrast without the use of ionizing radiation, which can potentially damage biological tissues. Compared to other imaging modalities, optical imaging techniques have several advantages, such as cost-effectiveness, real-time imaging capability, and ease of implementation. The basic principle of optical imaging is image formation according to the absorption or scattering of photons. In typical optical microscopes, the absorbed or scattered light beams form shadows in the resulting images, thus the transparency of the target specimen is visualized in resulting images. However, in biomedical optical imaging techniques such as fluorescence imaging (FLI) and two-photon microscopy (TPM), the relaxation of the absorbed light energy generates optical waves that are detected by optical sensors resulting in bright pixel values 13.

**Figure 1:**
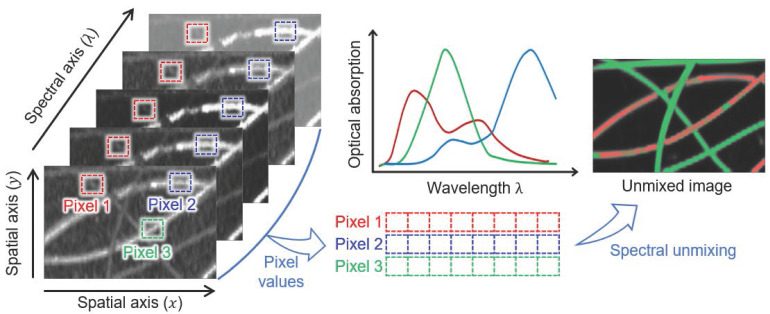
Schematic illustration of the spectral unmixing concept for the multispectral images. The images are reproduced with permission from Ref. 43

One major advantage of optical imaging is the ability to investigate molecular functional information from the multispectral imaging technique, which is the spectroscopy method acquiring both spatial (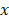 and 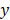 axes) and spectral (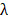 axis) information of biological tissue (Fig. 1). Image stacks are acquired by storing data under the illumination of various wavelengths of light to analyze the multispectral response. By comparing the pixel values at the same spatial position as a function of wavelength, we can delineate tissues with different spectral responses. Moreover, the composition of chromophores can be spectrally unmixed by mathematical calculation. The typical method to calculate the concentration of each chromophore is linear unmixing, which can be derived from the relationship that the measured signal is the weighted sum of the optical absorption coefficient of chromophores.


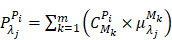

(1)

where 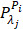 is the measured optical signal in the 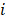-th pixel at the 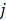-th wavelength, 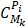 is the concentration of the 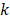-th chromophore in the 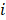-th pixel, 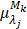 is the optical absorption coefficient of the 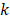-th chromophore at the 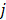-th wavelength, and 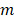 is the number of chromophores. The extended equation for all the pixels can be expressed as follows.


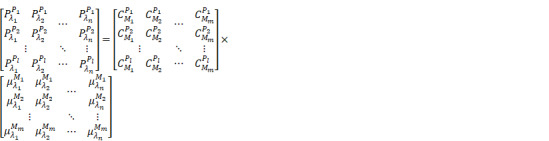

(2)

where 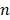 is the number of wavelengths and 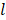 is the number of pixels in the image. Equation 2 can be simplified to the multiplication of matrices as follows, then the concentration of each chromophore in each pixel can be calculated through the pseudo-inverse operation. Note that the 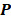 and 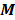 are measured values and 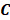 is the unknown parameter that we have the interest to calculate.


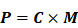

(3)


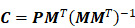

(4)

In addition to the linear unmixing, the model-based technique has also been investigated for spectral unmixing 14. In this technique, a model of spectral response is designed, then the amount of each component is calculated based on the model. Recently, deep learning technology has also been applied for the unmixing of the multispectral signal 15. By using those methods, individual signals of each chromophore can be extracted from the composite of multispectral responses.

Because the optical absorption and scattering characteristics are the composition of the components in biological molecules, the biological or pathological changes can be revealed and monitored using multispectral imaging and analysis 16-18. Therefore, the multispectral imaging techniques have widely applied and extended to biomedical studies for understanding the physiological response of biological tissues. In addition to the intrinsic chromophores, exogenous nanoparticles that generate optical contrast have widely been investigated for monitoring drug delivery, visualizing the biodistribution, and assessment of the therapy efficacy 19-21. Since the optical absorption spectra of the nanoparticles are typically measured before the injection, the multispectral analysis with spectral unmixing also can be performed. Although optical imaging techniques have advantages of multispectral analysis, the major drawback that limits themselves in clinical applications is shallow penetration depth. The strong light scattering in biological tissue makes it difficult to maintain the optical focus beyond the optical transport mean free path (i.e., ~1 mm). Therefore, optical imaging techniques are typically used in preclinical small animal studies 22-24.

To overcome the shallow imaging depth of optical imaging methods, photoacoustic imaging (PAI) has widely been investigated. The unique difference of PAI from other optical imaging techniques is that the signal is delivered as acoustic waves, which are scattered much less in biological tissue. To capture the acoustic waves, ultrasound (US) transducers are typically used in PAI 25. Compared to pure optical imaging, PAI can provide a deeper imaging depth of ~2-3 cm in soft tissue, while preserving the capability of multispectral analysis. Similar to the optical imaging techniques, multiple wavelengths of the excitation beams are used for multispectral analysis in PAI. Due to its relatively deep imaging depth, PAI has been applied to clinical studies 26-29 of breast cancers 30-32, thyroid cancers 33-36, prostate cancer 37, and melanoma 38, 39, as well as small animal studies 40-43.

Here, we review the multispectral imaging results of nanoparticles such that assessing molecular functional responses of biological systems *in vivo*. The principles of the typical multispectral imaging techniques (i.e., FLI, TPM, and PAI), which have been widely used for biomedical studies using nanomaterials, are summarized. By exploring the typical configurations, performance benchmarks, and representative biomedical applications of each technique, we highlight the advantages and limitations of multispectral imaging modalities. From the summary of the recent research articles which conducted multispectral analyses of nanomaterials *in vivo*, this article can provide future direction for biomedical imaging techniques to expand their application area.

## Principles and Implementation of Imaging Systems

**Figure 2:**
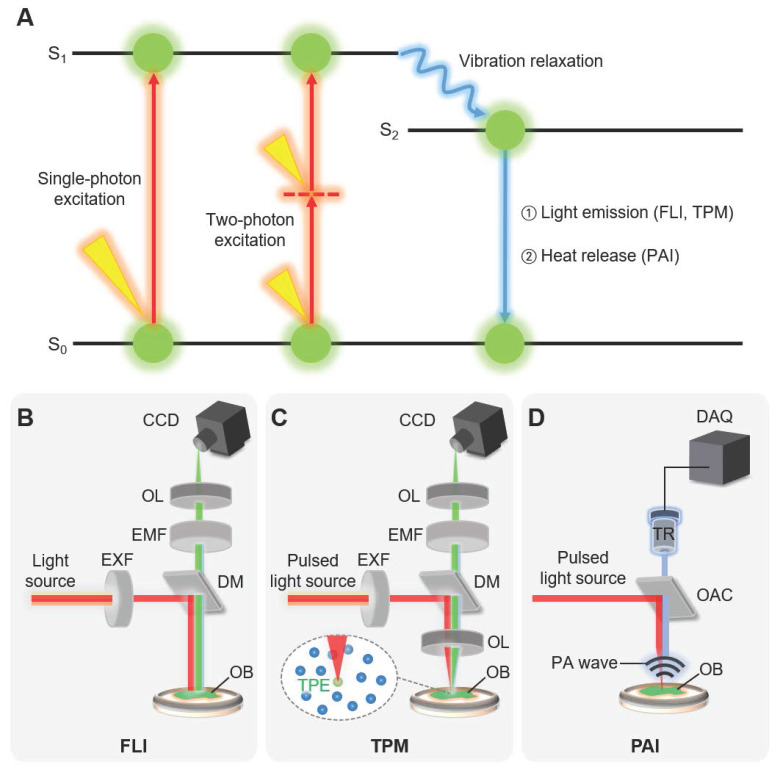
Principles and implementation of typical multispectral imaging techniques. (A) Schematic diagram of principles of signal generation. Red arrows depict the excitation of an electron by absorbing light energy, and blue arrows depict the release of the absorbed energy. (B-D) Schematic illustrations of typical FLI, TPM, and PAI systems. Inset in (C) depicts TPE at the focal point of the excitation laser. FLI, fluorescence imaging; TPM, two-photon microscopy; PAI, photoacoustic imaging; TPE, two-photon excitation; EXF, excitation filter; EMF, emission filter; OL, optical lens; DM, dichroic mirror; OB, target object; CCD, charge-coupled device; OAC, optoacoustic combiner; PA photoacoustic; and DAQ, data acquisition module.

The typical multispectral imaging techniques for nanomaterials (i.e., FLI, TPM, and PAI) share a similar principle of the imaging mechanism, which is absorption and emission of energy (Fig. 2A) 44-46. The energy is delivered in the form of light, illuminating the substance to be visualized. When the molecules in the ground state (S_0_ in Fig. 2A) absorb the photon energy, the electron in the outer shell is excited to the higher orbit, changes the state of molecules the excitation state energy level (S_1_ in Fig. 2A). The excited molecules return to the ground state again with a very short half-life time of typically a few nanoseconds. During this short period, the excited electron losses some amount of energy through vibration relaxation, generally dissipated as heat, then move to the lower state energy level (S_2_ in Fig. 2A). Consequently, the electron rapidly returns to the ground state by releasing its energy in the form of light (i.e., fluorescent light) or heat. Note that the wavelength of the emitted fluorescent (FL) light is longer than the wavelength of the excitation light. The wavelength of light can be expressed as the following equation.


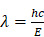

(5)

where 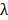 [m] is the wavelength of the light, 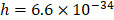 [Js] is Planck's constant, 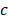 [m/s] is the speed of the light, and 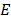 [J] is the quantum energy of the light.

FLI systems measure the intensity of the FL light emitted from the chromophores in biological tissue typically using charge-coupled device (CCD) cameras (Fig. 2B). To improve the contrast of the FL signal against the background signal, the fluorescence systems are generally designed so that the excitation and emitted lights pass through optical wavelength filters 47. The excitation filter that filters the wavelength of the excitation light is selected as close to the peak absorption wavelength of the chromophore. The emission filter refines the emitted FL light, such that only desired wavelengths are passed to the CCD camera. To prevent the excitation light from being detected, a dichroic mirror with an appropriate transition wavelength is used in the integrated FLI system.

TPM is based on the selective non-linear excitation of the chromophores in a particular focal region 48. Unlike FLI where a single photon is absorbed, two photons with half the energy (i.e., double the wavelength) from pulsed laser beams are used to excite the electron in TPM (two-photon excitation in Fig. 2A). The excitation of the molecules by the two photons is maximized at the focal region of the illuminated light (Fig. 2C). The FL light is emitted only at the focal region because the two-photon excitation is not high enough out of the focal region. Therefore, TPM can achieve 3D images by scanning the focal spot of the excitation light in 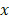, 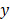, and 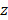 directions.

In contrast to FLI and TPM, PAI uses heat release for the image contrast instead of FL light (Fig. 2D) 49. The released heat makes the target molecules expand through thermoelastic expansion. Since PAI systems use short-pulsed laser beams, the heat is rapidly dissipated, and the expanded molecules shrink back to their original size. This rapid change creates thermoelastic expansion, then generates acoustic waves called photoacoustic (PA) waves 50. The generated PA waves propagate in all directions and can be detected by the conventional US transducers (Fig. 2C). The unique feature of PAI is the acoustic detection of optical absorption properties of target specimens. In addition to the less scattering of the acoustic wave, which enables PAI to be used in deep tissue imaging 51, 52, the relatively slower propagation speed (1540 m/s in soft tissue) leads to the depth-resolved signal detection and thus 3D imaging is capable without scanning in the 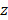 direction.

The main difference between FLI/TPM and PAI is the form of energy release: irradiation of FL light or acoustic wave generation through thermoelastic vibration. However, the two types of energy release are mixed rather than either. The efficiency that an excited electron will emit the FL light can be express as follow.


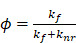

(6)

where 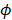 is the fluorescence quantum yield, 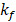 is the rate constant for radiative relaxation (i.e., rate of FL light emission), 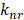 is the rate constant for non-radiative relaxation (i.e., rate of heat release). In general, exogenous nanoparticles that are used as the contrast agents for these imaging techniques do not have 100% or 0% efficiency. In other words, both PAI and FLI/TPM can be performed 53-57 by injecting a single contrast-enhancing nanoparticle that has appropriate 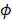 (Table 1) 58.

In addition to contrast-enhanced imaging, various functionalized nanoparticles have been investigated for visualization of biological conditions *in vivo* 59-61. By synthesizing with the targeting function, the nanoparticles can specifically interact with the target, thus can visualize functional procedures of biological systems, such as tumor growth 62-64, lymphatic drainage 65, and whole-body distribution of drugs 66.

## Multispectral Fluorescence Imaging of Nanoparticles

FLI with FL nanoparticles has been widely applied for monitoring and tracking the biodistribution of agents in real-time 67-69. In particular, the study of FLI with the excitation of near-infrared (NIR) light sources has been used for whole-body imaging since the photon scattering of NIR light is less than that in other wavelength regions. In general, the emitted fluorescence signals are mixed with the autofluorescence from the tissue, and it prevents obtaining clear images from the biological tissue. However, in multispectral FLI, it is possible to differentiate the FL signals of various probes by analyzing the response from different wavelengths. Recently, several studies using multispectral FLI had been conducted for preclinical and clinical applications including sensitive tumor labeling 70, monitoring drug delivery 71, 72, and image-guided surgery 73-75. Recently, several examples of *in vivo* multispectral FLI have been reported using FL nanoparticles.

**Figure 3:**
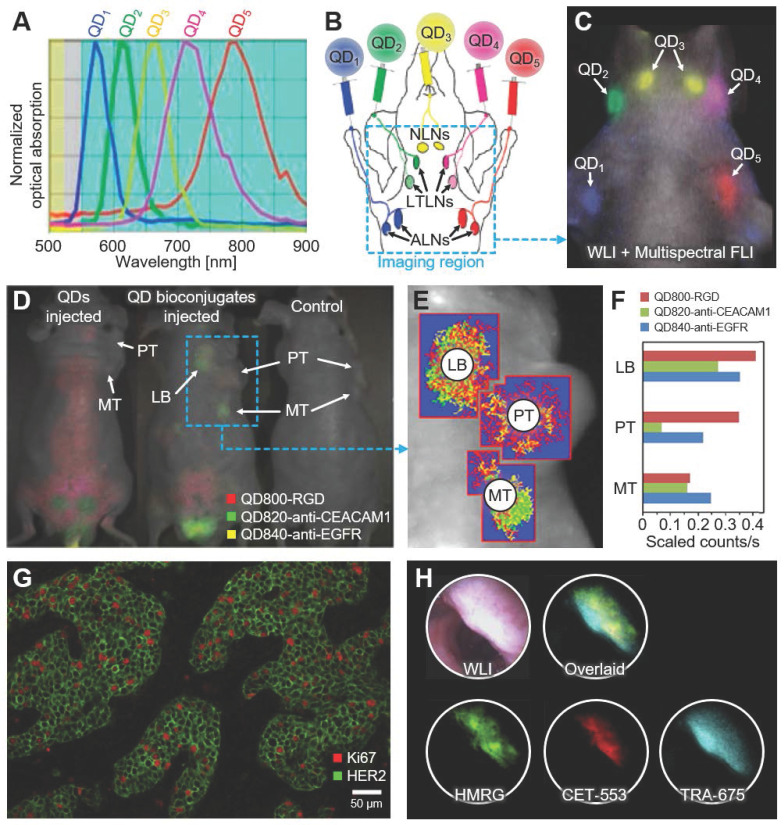
Representative results of multispectral FLI using fluorescent nanoparticles. (A) Emission spectra of five carboxyl QDs for contrast-enhanced lymph node imaging. (B) Schematic illustration of the injection site and the anatomy of the lymphatic system and the injection. Blue dashed box depicts imaging region. (C) The overlaid *in vivo* image of multispectral FLI and WLI after injection of the five carboxyl QDs. (D) The overlaid *in vivo* images of multispectral FLI and WLI from the human lung epithelial carcinoma xenografts in nude mice. (E) Region of interests for multispectral analysis. (F) Quantified unmixed signal in each region of interest. (G) Multispectral FLI of Ki67 and HER2 co-expressions in breast cancer cells. (H) *In vivo* WLI, multispectral FLI, and overlaid images of polyps using fluorescent probes. FLI, fluorescence imaging; WLI, white light imaging; QD, quantum dot; NLN, neck lymph node; LTLN, lateral thoracic lymph node; ALN, axillary lymph node; PT, primary tumor; MT, metastatic tumor; LB, lymphatic basin; RGD, arginine-glycine-aspartic acid; anti-CEACAM1, anti-carcinoembryonic antigen-related cell adhesion molecule-1; anti-EGFR, anti-epidermal growth factor receptor; HER2, human epidermal growth factor receptor-2. The images are reproduced with permission from Refs. 76-79.

At the early stage of multispectral FLI, Kobayashi *et al*. reported the multispectral FLI of lymphatic networks using five different NIR-FL probes 66. In the following study, they synthesized quantum dots (QDs) for the same purpose 76. Leveraging the nature of QDs whose absorption spectrum varies with size, they developed five different carboxyl-QDs with absorption peaks at 565, 605, 655, 705, and 800 nm, respectively (Fig. 3A). To validate the feasibility of contrast-enhanced imaging of QDs *in vivo*, they intracutaneously injected the five QDs into five different sites in mice (Fig. 3B). The multispectral FLI results showed that the five QDs were separately delivered to corresponding lymph nodes that matched with the anatomy of the lymphatic system in the upper body of the mouse (Fig. 3C). From the multispectral analysis, the accumulations of five QDs in the corresponding lymph nodes were successfully differentiated. The result showed the great potential of multispectral FLI to be used in multi-color lymphangiography.

In 2012, Han *et al.* introduced FLI-guided tumor detection and drug delivery using gold-doped CdHgTe QDs (Au:CdHgTe QDs) 77. They synthesized three different types of nanoparticles by conjugating arginine-glycine-aspartic acid (RGD) peptide, anti-epidermal growth factor receptor (anti-EGFR) monoclonal antibody (MAb), and anti-carcinoembryonic antigen-related cell adhesion molecule-1 (anti-CEACAM1) MAb separately. The three types of conjugates (i.e., QD800-RGD, QD820-anti-CEACAM1, and QD840-anti-EGFR) were simultaneously detected *in vivo*, especially showed great potential to be used for tumor targeting agents with specific delineation of tumor region (Fig. 3D). The multispectral FLI results verified the feasibility of the bioconjugated QDs for tumor targeting by comparing them with the FLI results of control mice. The mixtures of QDs were intravenously injected into human lung epithelial carcinoma xenografts in nude mice. In the results, the primary tumor, metastatic tumor, and lymphatic basin were delineated from the multispectral analysis of images (Fig 3E and 3F).

Xiang *et al.* proposed another QD-based nanoparticle that targets Ki-67 and human epidermal growth factor receptor-2 (HER2) 78. Since these antigens are expressed in large amounts during breast cancer manifestation, the malignant breast cancer cells could have been quantitatively studied by injecting the targeted nanoparticles. Figure 3G shows the multispectral fluorescence signals expressed on the surface of the target cells. Ki-67 was represented in red, while HER2 was in green. While the selection of FL probes is important for sensitive tumor detection and drug delivery monitoring, there was another study that has improved the accuracy of signal detection improving an endoscope system. Bae, *et al.* proposed a multispectral molecular imaging endoscopy system to acquire multispectral FLI of colon dysplasia affected areas, equipped with a liquid crystal tunable filter with a wavelength range of 420~720 nm 79. The system removes the autofluorescence of the lesion of interest by separating the multispectral signals, thus can successfully describe the colon dysplasia (Fig. 3H).

## Multispectral Two-Photon Microscopy of Nanoparticles

The most distinguishable feature of TPM compared to FLI is the two-photon excitation by light sources with longer wavelengths in the NIR region. Since the excitation occurs in the limited region of the focal point, which has a typical volume of a few femtoliters, TPM can achieve 3D images of high sensitivity and submicron resolution without using a confocal pinhole 80. In addition, deeper penetration of light in biological tissues (0.6 ~ 0.8 mm) is possible because of the reduced absorption and scattering of the longer wavelengths 81, 82. As two-photon emission is a non-linear process with a small cross-section, high photon flux is required to generate high efficiency in the process, which is typically achieved through ultrashort pulsed lasers. At each pulse of light, the number of photons absorbed in a sample is defined as follows 83.


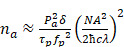

(7)

where 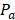 is the average power of the light source, 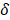 is the two-photon absorption rate of the fluorophore at the wavelength (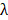), 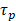 is the pulse duration of the light source, 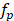 is the repetition rate of the light source, 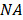 is the numerical aperture of the objective lens, 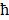 is Planck's constant, and 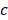 is the speed of light. From the equation, the shorter pulse duration leads to a higher probability of two-photon absorption when the average power is considered constant. Therefore, ultrashort (typically ~140 fs) pulsed laser has widely been used for TPM. The ultrashort laser pulse also reduces the photodamage to the biological sample, thus can be used for *in vivo* imaging of live samples. Moreover, multispectral TPM can identify the composition of biological tissues labeled with several FL nanoparticles. Recently, multispectral analyses of nanoparticles using TPM have been explored for applications including therapeutic process 84, 85, cell tracking optical probe 86-88, the morphogenetic process of neurons 89, 90, and functional analysis to reveal disease progression at the cellular level 91-93.

**Figure 4:**
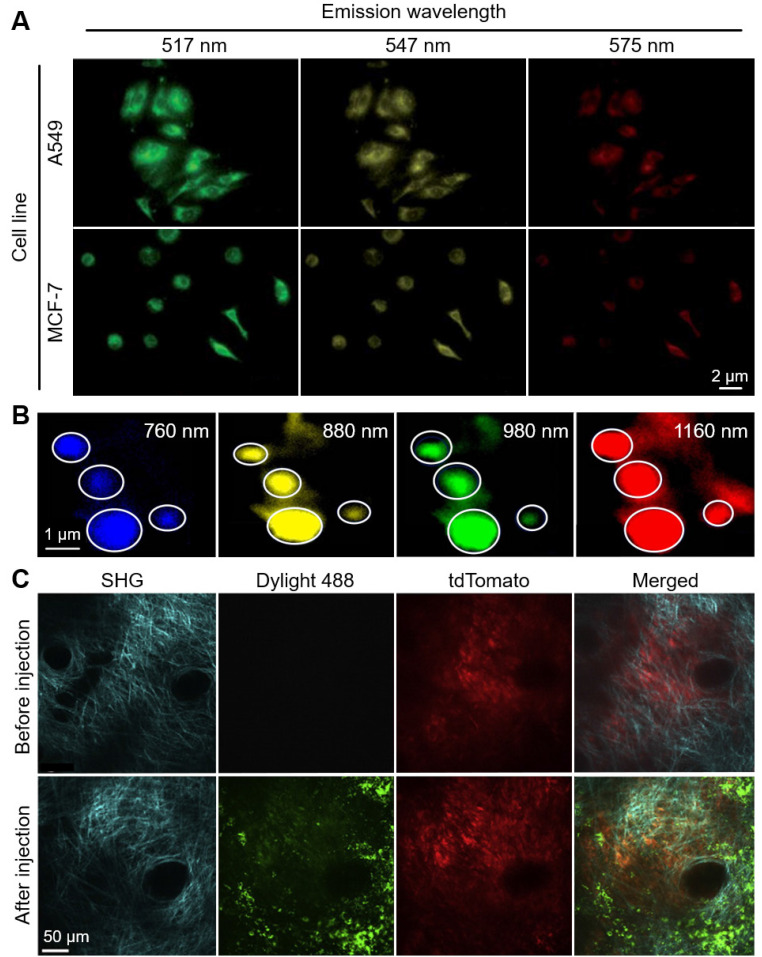
Representative results of multispectral TPM using FL nanoparticles. (A) Multicolor TPM of A549 and MCF-7 cells using multi-emission FL nanoparticles. (B) Multicolor TPM images of MRSA using aptamer-modified GO sheets with various excitation wavelenghts. White circles depict the positions of MRSAs. (C) TPM images of a xenografted melanoma in a mouse ear before and after injection of Dylihgt 488 conjugated nanoparticles. TPM, two-photon microscopy; FL, fluorescent; MRSA, methicillin-resistant *Staphylococcus aureus*; SHG, second harmonic generation. The images are reproduced with permission from Refs. 96, 98, 99.

FL nanoparticles also have widely been used in TPM. To achieve high efficiency of FL light emission, the nanoparticles are typically synthesized from the aggregated fluorophores because they have large stroke shifts caused by excited-state intramolecular proton transfer (ESIPT) molecules 94, 95. In 2014, Deng *et al.* demonstrated the two-photon absorption of FL nanoparticles derived from several salicylidenealiline molecules 96, which are well-known molecules that demonstrate the ESIPT phenomenon 97. They achieved multicolor TPM images of human alveolar basal epithelial (A549) and breast cancer (MCF-7) cells under the excitation wavelength of 405 nm (Fig. 4A). The results showed the feasibility of multispectral TPM as a promising platform for multicolor cell imaging applications.

Pramanik *et al.* reported an aptamer conjugated graphene oxide (GO), which can be used for multiple drug-resistance bacteria (MDRB) imaging 98. They found that the emission wavelength of the aptamer conjugated GO was tunable from visible to NIR by varying excitation wavelength, without changing its chemical properties or size (Fig. 4B). They demonstrated the multicolor TPM images of methicillin-resistant *Staphylococcus aureus* (MRSA) in the aptamer conjugated graphene oxide nanosheets. The nanoparticles have efficient two-photon emission with two-photon excitation through the wavelengths of 760, 880, 980, and 1160 nm. By demonstrating the high efficiency of two-photon excitation, photo-stability, and biocompatibility, the results showed the feasibility of multispectral TPM of GO nanoparticles for delineating the multiple MDRBs.

TPM also has been utilized for multicolor tumor imaging from the uptaken nanoparticles in tumor cells. Roode *et al.* achieved multispectral TPM images from a xenografted melanoma tumor in the ear dermis of mice 99. They used particle replication in the non-wetting templates (PRINT) technique to produce uniformly sized nanoparticles. They investigated the tumor uptake of the PRINT-nanoparticles by simultaneously acquiring TPM images of the tumor (tdTomato) and the FL nanoparticles (Dylight488). They also acquired the second harmonic generation (SHG) signal which can detect the signals from the bundled collagen (Fig. 4C). The results showed the potential of the multispectral TPM for analyzing the further in-depth correlation between the characteristics of nanoparticles and tumor cells.

## Multispectral Photoacoustic Imaging of Nanoparticles

In contrast to the pure optical imaging techniques, where the imaging depth is greatly limited by strong photon scattering in biological tissues, PAI allows deeper imaging depth because the signal is transported through acoustic waves that are less scattered in biological tissues compared to the photon. Although the signal is delivered as a form of the acoustic wave in PAI, the generation of the signal is based on the optical absorption, which enables PAI can be used for multispectral analysis by tuning the wavelength of the illumination light source. Several endogenous chromophores such as oxy-hemoglobin, deoxy-hemoglobin, melanin, and lipid, PAI can provide both functional and structural information of biological tissues 100-102. In addition to endogenous imaging, contrast-enhanced PAI has widely been investigated by using various exogenous nanoparticles with functionalities of contrast enhancement, disease targeting, or drug delivery 103-106.

**Figure 5:**
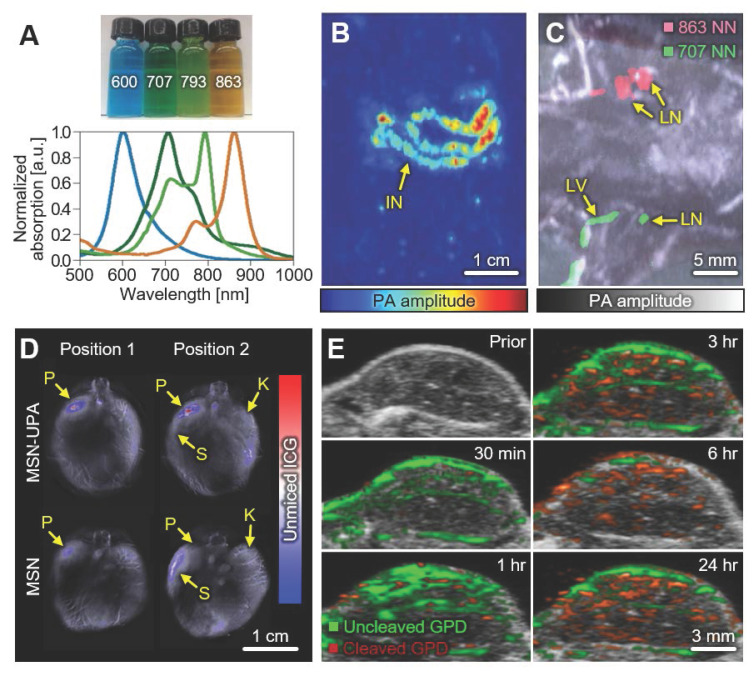
Representative results of multispectral PAI using contrast-enhancing nanoparticles. (A) Optical absorption spectra of four NNs for contrast-enhanced PAI. Numbers in the photograph represent the absorption peak of each NN. (B) Contrast-enhanced PA image of gastrointestinal tract of a NN-injected mouse *in vivo*. (C) Delineation of axillary lymphatic networks from *in vivo* multispectral contrast-enhanced PA images. (D) Unmixed ICG images showing nanoparticle accumulation in pancreatic tumor *in vivo*. (E) Multispectral PA images of glioblastoma after intravascular injection of GPD probes. PAI, photoacoustic imaging; PA, photoacoustic; NN, naphthalocyanine nanoparticle; LN, lymph node; LV, lymphatic vessel; ICG, indocyanine green; MSN, mesoporous silica nanoparticle; UPA. urokinase plasminogen activator; P, pancreas; S, spleen; K, kidney; GPD, gold nanocage-peptide-fluorescent dye. The images are reproduced with permission from Refs. 107-109, 111

Zhang *et al.* introduced naphthalocyanine nanoparticles (NNs) that have stable optical absorption spectra with controllable absorption peaks (Fig. 5A) 107. They showed the feasibility of contrast-enhanced PAI with an application of the gastrointestinal tract of mice *in vivo*. The NNs were injected by oral administration, then the distribution of the nanoparticles through the gastrointestinal tract was successfully visualized (Fig. 5B). In the following study, Lee *et al.* delineated multiple lymphatic paths by multispectral PA analysis of mice *in vivo* 108. They subcutaneously injected two different NNs (absorption peaks at 707 and 863 nm, respectively) at each side of the forepaw, acquired multispectral PA images, visualized lymphatic drainage by overlaying unmixed PA signals (Fig. 5C). The results show the potential of nanoparticles for multispectral analysis of contrast-enhanced PA images *in vivo*.

In 2016, Gurka *et al.* showed improved pancreatic tumor imaging using mesoporous silica nanoparticles (MSNs) 109. They added chitosan and urokinase plasminogen activator (UPA) receptors to the nanoparticles for targeting the acidic tumor microenvironment and overexpressed receptors in tumors, respectively. In addition, indocyanine green was loaded for multispectral PAI. They acquired multispectral PA images of pancreatic cancer (S2VP10 cell line) bearing mice *in vivo*, with wavelengths of 680, 710, 730, 740, 760, 770, 780, 800, 850, and 900 nm. The spectrally unmixed PA signals show that the UPA-added MSNs (MSN-UPAs) accumulates more in the pancreatic tumor compared to the pure MSNs (Fig. 5D). The results show the illustration of the targeting efficacy of nanoparticles, which can be utilized for a variety of studies in drug delivery and disease identification.

Over the past few decades, gold nanoparticles have gained much attention for contrast-enhanced imaging due to their excellent optical absorption, easiness of synthesis, and chemical stability 110. Liu *et al.* demonstrated the contrast-enhanced tumor images using an FL dye (Alexa Fluor Dye680) conjugated gold nanocages (GNCs), especially for tumor protease detection in 2018 111. Tumor protease is one of the important factors controlling tumor proliferation, thus it is useful to identify the degree of tumor activation. The synthesized GNC-peptide- FL dyes (GPDs) were targeted to the MMP-2 protease through the enzymatic peptide substrate. When the peptide substrate was cleaved after combining with MMP-2, a distinct chemical change of tumor protease signal was detected from glioblastoma xenografts in nude mice *in vivo* (Fig. 5E). As the time flows, the PA signals from the uncleaved GPDs (green in Fig. 5E) decreased while the PA signals from the cleaved GPDs (red in Fig. 5E) increased. The results show the feasibility of multispectral PA for monitoring chemical response in biological tissues *in vivo*.

## Summary and Conclusion

Multispectral imaging techniques are the methods that detect and classify features. The underlying concept of the technique is the spectral unmixing of the signals based on the spectral responses of chromophores. In this review, we summarized the basic principles of representative multispectral imaging techniques, then explored their applications in biomedical studies (Table 2). The contrast-enhanced images from the administration of nanoparticles enable the multispectral analysis to differentiate lymphatic networks, delineate diseased regions, and monitor the chemical changes in biological tissues. The representative results show the feasibility of the multispectral analysis of nanoparticles in the exploration of anatomy, biochemistry, pathology, and physiology in preclinical biomedical engineering. As potential clinical diagnosing tools, multispectral imaging systems have been used to monitor biological changes *in vivo* by delivering a variety of nanoparticles. To expand the application area to the clinical world, several challenges should be addressed in developing the nanoparticle including reducing toxicity, ensuring biocompatibility, enhancing biodegradability, and improving photostability. In summary, the multispectral analysis of nanoparticles has great potential to be used in a variety of preclinical and clinical biomedical researches. The continuous efforts by researchers to improve the system configuration for achieving a higher signal-to-noise ratio, a better spatiotemporal resolution, and a deeper imaging depth would expand the application areas of the multispectral imaging techniques. Therefore, the spectral imaging of nanoparticles would become an essential tool for biological and biomedical studies.

## Figures and Tables

**Table 1:** Optical characteristics of fluorophores that have been used for multimodal PAI and FLI/TPM. PAI, photoacoustic imaging; FLI, fluorescence imaging; TPM, two-photon microscopy; 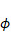, fluorescence quantum yield; PBS, phosphate-buffered saline; ICG, indocyanine green. The data are achieved and reproduced with permission from Ref. 58.

Fluorophore	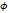 (in PBS)	Absorption [nm]	Emission [nm]	Ref.
ICG	0.042	777	802	54, 55
Alexa Fluor 750	0.12	749	775	56
IRDye 800CW	0.12	774	789	57

**Table 2:** Performance benchmarks and biomedical applications of imaging systems that used multispectral analysis of nanoparticles. fluorescence imaging; TPM, two-photon microscopy; PAI, photoacoustic imaging. N/A, not available.

Ref	Imaging modality	Model	Spatial resolution	Imaging depth	Imaging speed	Applications
76	FLI	Maestro(CRi)	25 μm	N/A	10 sec	Lymph node mapping
77	FLI	Maestro(CRi)	25 μm	N/A	10 sec	Whole-body distribution mapping
78	FLI	Nuance(CRi)	~1 μm	N/A	~5 sec	Breast cancer cell imaging, Cell identification
79	FLI	Nuance(CRi)	~1 μm	N/A	~5 sec	Endoscopic imaging of polyps in mice colon
96	TPM	N/A	N/A	N/A	N/A	Breast cancer cell imaging, Cell identification
98	TPM	FV1000MPE(Olympus)	~1 μm	320 μm	16 fps	Aggregation monitoring of MRSA bacteria in aptamer
99	TPM	FV1000MPE(Olympus)	~1 μm	320 μm	16 fps	Melanoma imaging,Particle uptaken assessment
107	PAI	Custom 41	590 μm	~10.3 mm	~20 min	Gastrointestinal track monitoring,Detection of small bowel obstraction
108	PAI	Custom 41	590 μm	~10.3 mm	~20 min	Lymph node mapping
109	PAI	inVision 256TF(iThera Medical)	150 μm	~10 mm	10 fps	Accumulation monitoring of particles in pancreatic tumor
111	PAI	Vevo2100 LAZR(VisualSonics)	N/A	~5 mm	20 fps	Accumulation monitoring of particles in glioblastoma
